# Concept and proof for an all-silicon MEMS micro speaker utilizing air chambers

**DOI:** 10.1038/s41378-019-0095-9

**Published:** 2019-10-07

**Authors:** Bert Kaiser, Sergiu Langa, Lutz Ehrig, Michael Stolz, Hermann Schenk, Holger Conrad, Harald Schenk, Klaus Schimmanz, David Schuffenhauer

**Affiliations:** 1 Fraunhofer-Institute for Photonic Microsystems, 01109 Dresden, Germany; 2 Chair of Micro and Nano Systems, Brandenburg University of Technology Cottbus-Senftenberg, 03013 Cottbus, Germany

**Keywords:** Electrical and electronic engineering, NEMS

## Abstract

MEMS-based micro speakers are attractive candidates as sound transducers for smart devices, particularly wearables and hearables. For such devices, high sound pressure levels, low harmonic distortion and low power consumption are required for industrial, consumer and medical applications. The ability to integrate with microelectronic circuitry, as well as scalable batch production to enable low unit costs, are the key factors benchmarking a technology. The Nanoscopic Electrostatic Drive based, novel micro speaker concept presented in this work essentially comprises in-plane, electrostatic bending actuators, and uses the chip volume rather than the its surface for sound generation. We describe the principle, design, fabrication, and first characterization results. Various design options and governing equations are given and discussed. In a standard acoustical test setup (ear simulator), a MEMS micro speaker generated a sound pressure level of 69 dB at 500 Hz with a total harmonic distortion of 4.4%, thus proving the concept. Further potential on sound pressure as well as linearity improvement is outlined. We expect that the described methods can be used to enhance and design other MEMS devices and foster modeling and simulation approaches.

## MEMS micro speakers: Speaking volumes

A new concept for an all-silicon CMOS compatible microspeaker uses the volume of the chip to generate sound. Microspeakers are used in a range of smart devices, and must provide both high sound pressure levels and low harmonic distortion. The majority of existing devices achieve this by out-of-plane deflection of surfaces, such as membranes or plates. Now, a project run by Bert Kaiser from Fraunhofer-Institute for Photonic Microsystems demonstrates micro speakers that comprise of in-plane electrostatic bending actuators that utilize the volume of the chip to generate sound. They first introduce the concept of their device and then demonstrate a working system, achieving a sound pressure level of 69 dB at 500 Hz and a total harmonic distortion of 4.4%.

## Introduction

There is great demand for high performance micro electromechanical systems (MEMS) micro speakers by the consumer and by the hearing aid industry. Key factors, such as high sound pressure level (SPL), low total harmonic distortion (THD), low power consumption, small device footprint and batch fabrication possibilities are needed to outperform classic micro speaker technologies like moving coil and balanced armature receiver technology. MEMS-based micro speakers achieve all these key factors. Successful devices made with MEMS, like inertial sensors, timing devices and pressure sensors, are ubiquitous in many electronical applications. These applications range from vehicles to wearables. A prominent example is smartphones, which have all the types of MEMS mentioned above. They also include acoustic transducers, most likely appearing as multiple MEMS microphones, to aid their original primary application, voice communication. In contrast to fabrication, pricing and integration logic, today’s smartphones include fine mechanical engineered, non-MEMS micro speakers. Hearables, which are wearables plugged directly into the ear canal and relying mostly on acoustics for their human-machine interface, are expected to take over the acoustic functionality of smartphones and extend their application to that of personal assistant. In earbuds, the available space is limited, thus demanding exceptional miniaturization and integration of all components including electronics, sensors, batteries, and acoustic transducers.

Several concepts for MEMS-based micro speakers have been reported in the literature. Among them are the following examples. Cheng et al.^1^ published an electromagnetically driven MEMS micro speaker comprising an electroplated, micro machined membrane with a diameter of 3.5 mm and a permanent magnet assembled underneath. The device reached 93 dB at 5 kHz in a 2 cm^3^ closed cavity, consuming 320 mW of power. No results for the THD were published. Chen et al.^2^ used a 3.5 mm diameter polymer membrane on a silicon substrate, incorporating a single loop copper coil and two magnets for electromagnetic driving of the speaker. They were able to demonstrate 106 dB at 1 kHz in a 2 cm^3^ closed cavity, with an input power of 1.76 mW. No values for the THD were reported. Albach et al.^3^ published the concept of a MEMS-based micro speaker driven by a changing magnetic field. The concept describes a parallel arrangement of bending actuators, using a magnetostrictive active layer with a photoresist passive layer. The actuators form an acoustically closed membrane of about 7.5 mm^2^, despite the gaps between the individual cantilevers. In a hybrid setup, Albach et al. drove the MEMS devices with externally generated magnetic fields. A SPL 101 dB was measured in a 2 cm^3^ closed cavity at 400 Hz. No values for either the THD or power consumption were presented. A sound transducer based on the piezoelectric bending of actuators was presented by Stoppel et al.^4^. They describe a concept where the laterally expanded, triangular actuators deflect out-of-plane according to the bimorph effect. For a driving voltage of 2 Vpp they were able to measure a peak SPL of 86 dB (2 cm^3^ closed cavity) with a THD of less than 2% including a preprocessed input signal. A sensitivity of more than 105 dB/mW was achieved. Roberts et al.^5^ presented the concept of an electrostatically actuated MEMS micro speaker. It features a silicon carbide membrane on a silicon back-plate with an 8 µm electrostatic gap. They were able to measure a SPL of 73 dB at 16.6 kHz at a distance of 10 mm in free field radiation with a driving voltage of 200 Vpp. From the deflection and active area data given, this equals an approximate SPL of 65 dB in a closed 2 cm^3^ cavity. Neither the THD nor the power consumption data were reported. Fei et al.^6^ presented a thermoacoustic device based on graphene foam. The device exhibits an active area of 100 mm^2^. An SPL of 75 dB at 10 kHz in a free field measurement at 3 cm is reported for an input power of 1000 mW. This translates to approximately 84 dB in a 2 cm^3^ cavity. For the sake of completeness it should be noted that several works have been conducted with parametric transducers, utilized in arrays^7^, for digital sound reconstruction^8–11^. These approaches, which are presented in literature, are assumed to be relatively large because of the large number of single membrane drivers arranged in parallel. This renders the devices not beneficial for miniaturization in the first place.

The literature overview reveals various mechanical approaches, with at least three different actuating mechanisms. Except for the thermoacoustic devices, they all actuate single or multiple membranes or similar working entities which deflect out-of-plane. These membranes or plates exhibit different sizes and shapes and are sometimes closed or slitted.

Highly miniaturized MEMS concepts which do not require complex or elaborate assembly technologies, but rather allow the use of broadly available manufacturing technologies (are compatible with complementary metal-oxide semiconductor (CMOS) technology) are highly favored for scalable mass production.

In this paper we present a novel design concept for MEMS micro speakers based on the fabrication technologies referred to as silicon bulk micro machining, which is fully CMOS compatible. The design comprises all-silicon electrostatic, in-plane bending micro actuators working in air chambers, thus utilizing the chip’s bulk volume for air displacement and sound pressure generation. This micro speaker design approach allows the substitution of chip area for volume. In this work governing principles and parameter relations are presented and the concept’s potential is estimated. By fabricating and characterizing fabricated specimen, the concept is proven. To validate a concept, performance in terms of attainable sound pressure has to exceed the hearing threshold and should at least reach sound pressure values of normal speech, e.g., 55 dB at 1 kHz, and surpass previously reported values for electrostatic transducers^5^. Distortion in terms of THD should be below 10% and more likely below 5% as indicated by listening test^12^.

## Results

**Fig. 1:**
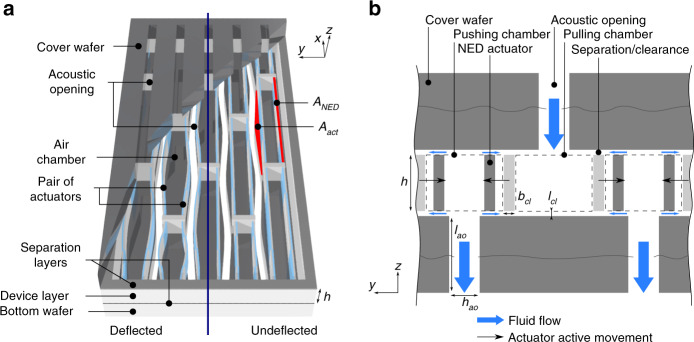
MEMS micro loudspeaker utilizing air chambers. **a** schematic 3D representation. **b** Cross-sectional schematic (not to scale)

The nanoscopic electrostatic drive (NED) based micro speaker concept is shown in Fig. 1a. The device comprises clamped-clamped electrostatic bending actuators, the NED^13^, placed pairwise in rows and columns within the device layer of a bonded silicon on insulator (SOI) wafer and covered by another wafer bonded to the SOI wafer with a tiny separation. Between each neighboring row of actuators, acoustically effective openings are integrated in the top and bottom of the wafer in an alternating way to allow the emission of sound from the device without acoustic shortcut.

The clamped-clamped configuration, when based on a suitable design, gives an efficient double-S bending shape to the NEDs. Every other row of paired actuators is shifted along the longitudinal axis by half the length of one actuator to make denser packing of the actuators possible whilst considering the bending shape. When a driving voltage is applied across the electrodes of the individual but identical actuators, they bend laterally in-plane according to their orientation in an odd mode relative to each other, thus balancing inertial forces within the device. With the top and bottom wafers as closing covers, each space between any of the actuators creates an air chamber. The function of each chamber at any one moment is to push air out into or to pull air in from the surroundings, depending on the direction of its associated actuators’ active deflection.

As a consequence of the deflection of the actuators, a fraction of the air volume Δ*V*_act_ of a pushing chamber is transferred out of the device through the associated acoustic opening. The same quantity of air is transferred into the device, i.e., into the pulling chambers. When the driving voltage is turned off, the restoring mechanical forces of the actuator beam bring it back to its rest position and invert the previous volume flow. Therefore, each pushing chamber becomes a pulling chamber and vice versa. This process may be driven at any frequency *f* in the audible range, thus generating sound. Considering inertial forces in a dynamic actuation regime the NEDs deflection may also get inverted, i.e., deflection passes beyond the mechanical rest position. A fact to be considered in the device design. The actuators must not pass the acoustic opening aperture in any operational state, as this will cause loss of sound pressure due to acoustic shortcutting.

The tiny separation between the in-plane deflecting actuators and the top and bottom wafer ensures movability of the actuators (see Fig. 1b). However, the separation represents a fluidic path between oppositely pressured chambers, thus enabling an acoustic shortcut. Geometrically, the clearance should be designed in such a way that losses are negligible, most likely by minimizing the distance between the actuators and the covers. In contrast, electrostatic forces generated by fringing electrostatic fields around the actuator tend to deflect the actuators in the z-direction, closing the clearance, and are reduced by a larger clearance. A vertical movement in the z-direction must be considered, since perfect symmetry of the upper and lower clearances is not to be expected in practice. Since the vertical stiffness of an actuator is proportional to *l*^*-*3^, the size of the clearance limits the viable actuator length *l*. If not designed properly, the vertical movement ultimately results in pull-in if the voltage exceeds the critical value *U*_pi_. This snap-in effect^14^ would create undesired friction between the actuator and the bottom or cover wafers, resulting in distortion (“rub & buzz”^15^) and substantially deteriorated sound quality. Furthermore, vertical pull-in may also result in electrical shortcutting and may lead to device failure.

The height of the actuators equals the SOI layer thickness and specifies the displaced volume for a given lateral actuator displacement. In a closed acoustic emission cavity, the sound pressure is proportional to the quotient of the volume change and the initial volume of the cavity. Thus, a given layout may be extended to an increased height into the SOI layer, thereby increasing the generable sound pressure without affecting the footprint of the device. This is a unique characteristic of the micro speaker concept presented in this paper and is in contrast to any other approach relying on membranes. The design possibilities, some of which have already been described above, comprise actuator design, actuator arrangement, actuator count, fluidic channels and damping entities. These all further shape the uniqueness of the concept. Nevertheless, the various layout options result in a rather complex design process and must be closely aligned with the technological boundary conditions, most likely relating to minimal widths and lengths, to exploit the full potential of the concept. To estimate this potential, fundamental aspects are presented in the following section.

### Theoretical description

Attainable sound pressure is the prominent performance indicator for micro speakers. In the following section, the limits are estimated using a geometric approach. For a given SOI layer thickness *h*, the acoustic performance is then specified by the arrangement and number of actuators, each exhibiting a certain geometry and area consumption *A*_NED_. While being displaced, the actuators sweep across an area *A*_act_, thus defining the ratio *r*_area_ = *A*_act_/*A*_NED_. The area *A*_act_ of the double-S bending shape may be simply calculated from the actuators’ maximum deflection at its middle yielding *A*_act_ = *w*_max_ * *l*/2. The area of the whole device *A*_dev_ equals the sum of the area of the actuators, considered a passive area, and the actively swept area, i.e., *A*_dev_ = *A*_NED_ + *A*_act_ = *A*_NED_ * (1 + *r*_area_). Comparing the actively scanned area to the device area defines the ratio *r*_fill_ = *A*_act_/*A*_dev_ = 1/(1 + 1/*r*_area_). Essentially, *r*_fill_ is in the range 0 < *r*_fill_ < 1 and is practically limited to *r*_fill_ << 1 because additional areas are required within *A*_dev_, such as for mechanical anchors and fluidic or electrical paths, as well as areas related to technological constraints. These areas may be subsumed into *A*_NED_ for the sake of simplicity.

The SPL (symbol: *L*_p_) in a closed acoustic emission space of volume *V*_0_, such as with earphones plugged into the ear canal, is not dependent on the position within this volume. Assuming wavelengths twice as big as the largest geometric dimension of *V*_0_ and that *V*_0_ is without leakage, the SPL can be simply calculated as follows. First, the pressure increase Δ*p* is calculated as:

1 $${\mathrm{\Delta }}p = \kappa p_0{\mathrm{\Delta }}V/V_0$$

where κ, *p*_0_ and *V*_0_ are the adiabatic index, the initial pressure of air and the volume of the acoustic cavity, respectively. The total displaced volume Δ*V* is the sum of all the volume changes caused by the single actuators, Δ*V*_act_. The SPL is then calculated as the logarithm of the pressure increase relative to an acknowledged referential pressure *p*_ref_:

2 $$L_p = 20\log \left( {\frac{{{\mathrm{\Delta }}p}}{{p_{\mathrm{ref}}}}\frac{1}{4}\sqrt 2 } \right)$$

The sqrt(2)/4 factor accounts for the RMS calculation of the sound pressure, assuming a harmonic sinusoidal movement of the actuators. Substituting Δ*V* = *A*_act_ * *h* = *A*_dev_ * *r*_fill_ * *h* gives

3 $$\begin{array}{l}L_p = 20\log \left( {\frac{{\kappa p_0}}{{p_{\mathrm{ref}}}}\frac{{A_{\mathrm{act}}h}}{{V_0}}\frac{1}{4}\sqrt 2 } \right)\\ L_p = 20\log \left( {\frac{{\kappa p_0}}{{p_{\mathrm{ref}}}}\frac{{A_{\mathrm{dev}}r_{\mathrm{fill}}h}}{{V_0}}\frac{1}{4}\sqrt 2 } \right)\end{array}$$

**Fig. 2:**
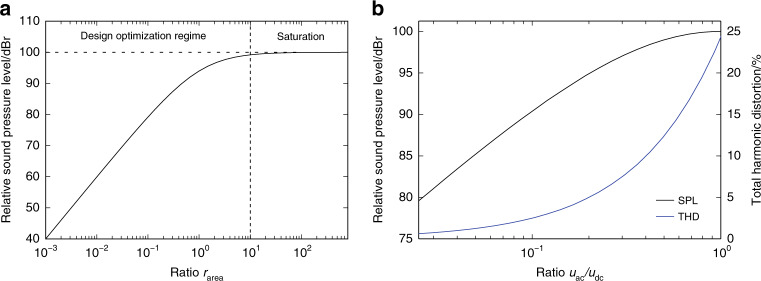
Sound output dependencies on geometry and electrical driving. **a** Dependency of a relative sound pressure level (arbitrary reference) as a logarithmic measure versus design factor *r*area. **b** Relative sound pressure level and THD (static domain) versus driving regime *u*_ac_/*u*_dc_ for *u*_dc_ + *u*_ac_ = const

Obviously, the maximum SPL is attained when *r*_fill_ = 1 and when device area and SOI thicknesses are maximized. This implies that the ratio *r*_area_ has a practically useable limit of about *r*_area_ = 10, giving an *r*_fill_ of approximately 9/10. In other words, there is no interest in actuators which exhibit a deflection greater than ten times their associated lateral dimensions. A ratio *r*_area_ > 10 would not increase attainable sound pressure significantly. Hence, the regime with values of *r*_area_ < 10 is referred to as the design optimization regime. Values of *r*_area_ > 10 are in the saturation regime. Figure 2a plots the attainable SPL against *r*_area_, showing the different regimes. The SPL is normalized using arbitrarily chosen parameters *A*_dev_, *h* and *V*_0_ so that the maximum achievable sound pressure for *r*_fill_ = 1 and *r*_area_ ≫ 100, respectively yields 100 dB, since it is a relative measure. Additionally, SPL may be estimated for different measurement cavity volumes using Eq. (3).

The previous SPL calculation assumes that the fluid transfer in and out of the device is through large enough openings which do not lead to, e.g., significant damping in the audible frequency range. Another important aspect is the split of fluid flows within the device, i.e., the differences in flow resistance between different fluidic paths. In the following, a static analysis is given. The flow resistivity of the acoustic openings may be estimated from the law of Hagen-Poiseuille^16^:

4 $$\dot V_{\mathrm{ac}} = K \ast \min \left( {b_{\mathrm{ao}},h_{\mathrm{ao}}} \right)^3 \ast \frac{{\max \left( {b_{\mathrm{ao}},h_{\mathrm{ao}}} \right)}}{{12\eta l_{\mathrm{ao}}}}{\mathrm{\Delta }}p_{\mathrm{ao}}$$

where *b*_ao_, *h*_ao_ and *l*_ao_ are the width, height and length of the acoustic opening channels respectively. It should be noted that the fluidic flow is in the direction parallel to the length of the opening channel *l*_ao_. The viscosity of air is encoded in ή. The correction factor *K* may be estimated as follows^16^:

5 $$K = 1 - \mathop {\sum}\limits_{n = 1}^\infty {\frac{1}{{\left( {2n - 1} \right)^5}} \cdot \frac{{192}}{{\pi ^5}} \cdot \frac{{\min \left( {b,h} \right)}}{{\max \left( {b,h} \right)}}\tanh \left( {\left( {2n - 1} \right)\frac{\pi }{2}\frac{{\max \left( {b,h} \right)}}{{\min \left( {b,h} \right)}}} \right)}$$

The pressure amplitude Δ*p*_ao_ may be derived from Eq. (1), taking *V*_0_ as the volume of one pushing or pulling chamber within the device (refer to Fig. 1b) and substituting Δ*V* with two single actuator displaced volumes Δ*V*_act_, so that Δ*p*_ao_ ∝ 2 Δ*V*_act_. The fluidic losses through the clearance, the rectangular opening along the actuator, may also be estimated. Two clearances of similar geometry are active for each actuator, with the Δ*p* being doubled because they have opposite signs on either side of the actuator, giving Δ*p*_cl_ ∝ 2 Δ*V*_act_. In contrast to the acoustic openings, a high fluidic flow resistance is desired for the clearance channels. The clearance channel has length *l*_cl_, width *b*_cl_ and height *h*_cl_. According to a typical layout, it is assumed that *b*_ao_ = *b*_cl_, *h*_ao_ ≫ *h*_cl_ and *l*_ao_ ≫ *l*_cl_ holds true. The ratio between the flow resistances of the clearance and acoustic openings may then be simplified as follows:

6 $$\frac{{\dot V_{\mathrm{cl}}}}{{\dot V_{\mathrm{ao}}}} \propto 2\frac{{h_{\mathrm{cl}}^3}}{{h_{\mathrm{ao}}^3}} \ast \frac{{l_{\mathrm{ao}}}}{{l_{\mathrm{cl}}}} \ll 1$$

Thus, the fluidic flow around the actuators through the clearance is significantly lower than through the acoustic openings. Consequently, no reduction in the acoustically effective volume flow must be assumed because of the air chamber concept.

The SPL described before defines the objective loudness of the acoustic transducer, i.e., acoustic quantity. In contrast, the THD defines the acoustic finesse, i.e., the acoustic quality of an acoustic transducer, making it an important measure for comparing sound reproduction. The THD shall therefore be discussed briefly and theoretically estimated in the following.

The working principle of the NED transducer is based on Coulomb forces with a quadratic dependence on the driving voltage as well as on the inverse of the electrode distance (Coulomb nonlinearity), which diminishes with increasing voltage^13^. The latter aspect is greatly influenced by the mechanics defined through the layout and how this affects the electrodes’, and consequently the actuators’, movements. For a clamped-clamped actuator configuration as given in our approach, a stress, resulting from deflection and inducing stiffening, i.e., nonlinear mechanical behavior known as the Duffing effect^17^, is to be expected. This effect develops contrarily to the previously mentioned electrode separation nonlinearity with increasing deflection of the whole actuator. Therefore, the focus is on the quadratic proportionality on the driving voltage only (a simplification). Adding an offset voltage *u*_dc_ is commonly known to enhance linearity, i.e., to decrease the distortion. Thus, the driving voltage is:

7 $$U = u_{\mathrm{ac}}\cos \left( {\omega t} \right) + u_{\mathrm{dc}}$$

The THD (symbol *k*; according to International Electrotechnical Commission IEC) is defined as the sum of all power _*pi*_ radiated in frequencies other than the desired excitation frequency, relative to the total emitted sound power.

8 $$k = 100\% \sqrt {\frac{{\mathop {\sum }\nolimits_{i = 2}^n p_i}}{{\mathop {\sum }\nolimits_{i = 1}^n p_i}}}$$

The actuator force *F* and deflection *x*, and thus its sound generation capability, are proportional to the square of the driving voltage *U*:

9 $$F\left( t \right)\sim \left( {u_{\mathrm{ac}}\cos \left( {\omega t} \right) + u_{dc}} \right)^2 = \left( {\frac{{u_{\mathrm{ac}}^2}}{2} + u_{\mathrm{dc}}^2} \right) + 2u_{\mathrm{ac}}u_{\mathrm{dc}}\cos (\omega t) + \frac{{u_{\mathrm{ac}}^2}}{2}{\mathrm{cos}}(2\omega t)$$

The average radiated power _*pi*_ is then proportional to the square of the signal:

10 $$\overline P \propto \frac{1}{T}\mathop {\int}_0^T {{\mathrm{d}}t} \,x^{\mathrm{2}}(t)\quad {\mathrm{with}}\,x \propto F$$

Comparing the amplitudes in Eq. (9), *k* reveals its dependency as follows:

11 $$k = 100\% \sqrt {\frac{{u_{\mathrm{ac}}^2}}{{u_{\mathrm{ac}}^2 + 16u_{\mathrm{dc}}^2}}} \quad {\mathrm{with}}\,|u_{\mathrm{ac}} + u_{\mathrm{dc}}| < U_{pi}$$

Because of the voltage limits due to pull-in, the sum of offset voltage and signal voltage need to be smaller than the pull-in voltage *U*_pi_ at any time. Therefore, the ratio *u*_ac_/*u*_dc_ needs to be minimized to minimize the THD. The SPL is dominated by the proportionality of *u*_dc_ and *u*_ac_. The SPL is then maximal for *u*_ac_/*u*_dc_ = 1. In Fig. 2b, SPL and THD are plotted against the ratio *u*_ac_/*u*_dc_. From this, a ratio *u*_ac_/*u*_dc_ < 1, e.g., *u*_ac_/*u*_dc_ = 1/8, seems reasonable to get low distortion of around 3% and a relative SPL of −8 dB (factor 0.4). For real devices, the SPL and THD follow more complex dependencies, including the mechanical nonlinearities mentioned above and fluidic interactions, e.g. from the acoustic load.

### Transducer design

**Fig. 3:**
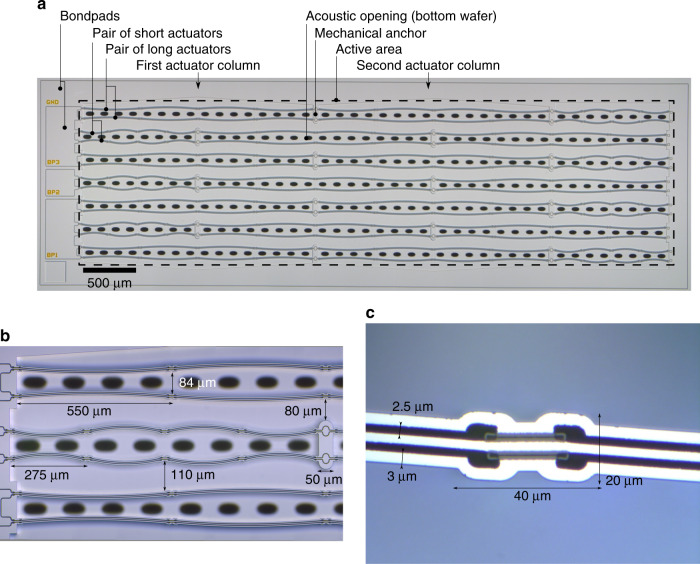
Geometric setup of fabricated MEMS speaker **a** Top view of the device layer showing actuators and their arrangement. Sample was taken out during fabrication without the cover wafer being bonded. **b** Enlarged partial view of three rows of actuators. **c** Detail of an actuator including dimensions

A first design was created and fabricated. The layout of the SOI layer is shown in Fig. 3a. It shows an arrangement of 14 pairs of long actuators in a 2-serial-7-parallel (2s7p) configuration, meaning that there are two actuator pairs in a row, and seven rows aligned parallel to the device plane. The actuators are bundled into three individually addressable groups (3-1-3 rows). To increase the fill factor of the design, remaining space to the edge of the preferred rectangular shaped device is filled with actuators of half the length. This adds seven pairs of half-length actuators in total. The footprint of the rectangular active area is 9.3 mm^2^. Each of the double clamped actuators has a length of 2.2 mm and 1.1 mm respectively. Vertical pull-in for the maximum driving voltage of 48 V is safely avoided, even after taking fabrication variations into account. The NED actuators’ shape was designed with finite element analysis-based optimization with the figure of merit being the displacement achievable while respecting technological boundary conditions (electrode thicknesses, trench widths, material properties). Details of the actuator arrangement and design properties are shown in Fig. 3b, c. The approximate width of the actuators is less than 20 µm. Each pair of actuators exhibits a distance of about 84 µm while each row of actuators is placed at about 110 µm from each other.

The device layout incorporates large (250 × 250 µm^2^) bond pads for wire bonding afterwards. Acoustic openings in the bottom and cover wafers are designed as rectangles located between every other actuator pair. The size of the opening is determined by the technological boundaries for etching, which defines their minimal width (30 µm). The bottom and top wafers are 400 µm thick. The distance (clearance) between the actuator and the bottom wafer equals the thickness of the 1 µm buried oxide layer of the bonded SOI wafer. This is also the design value for the separation to the top wafer, which can be defined by a deposited and patterned bonding interface layer. The SOI layer thickness is 75 µm.

Three different transducer devices were fabricated on one single chip. Fabrication was performed on silicon wafers, solely using CMOS compatible processes. The main process is deep reactive ion etching (DRIE). Details of the fabrication are supplied in the methods and materials section.

### Measurement setup and results

**Fig. 4:**
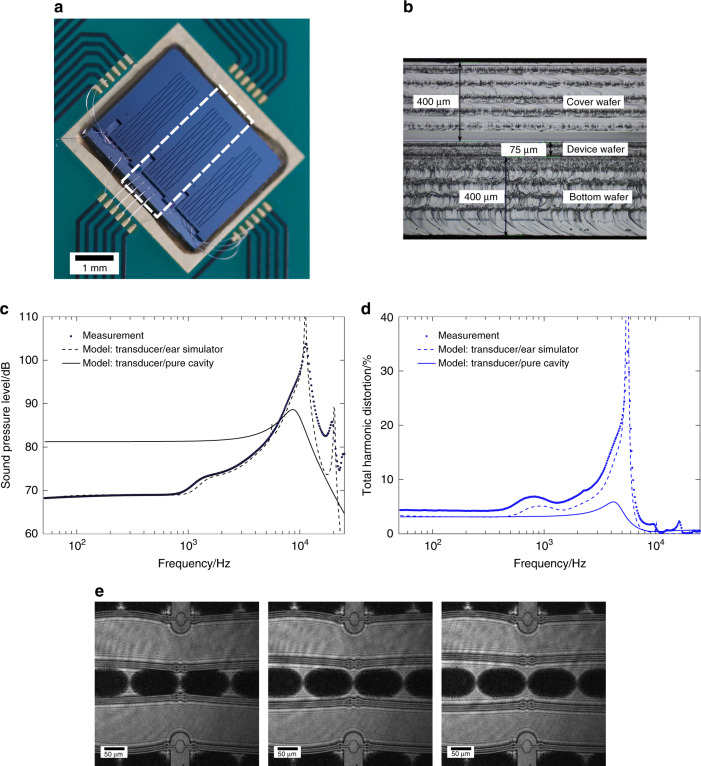
MEMS device setup and acoustical as well as optical measurement data **a** Assembled device with cover wafer on top showing its acoustic openings; the MEMS chip is glued to a carrier board enabling wire bonds for electrical contacting. The chip actually comprises three different micro speakers, which were not diced for this test set-up. The middle one was used as shown in (**a**). **b** Side view of the bonded wafer stack showing the three wafers and their thicknesses. Scallops resulting from cleavage through lacer dicing are clearly visible. **c** Measurement and modeling results for the SPL. Device was driven with *u*_dc_ = 40 V and *u*_ac_ = 10 Vpp. An SPL of 69 dB was achieved at 500 Hz. **d** THD measurement and modeling. THD value at 500 Hz is 4.4%. **e** Sequence of stroboscopic pictures from the holographic microscope showing three consecutive (time wise) deflections states in resonance of one actuator pair (specimen without cover wafer)

To characterize the acoustic performance, SPL and harmonic distortion tests were performed. The bare MEMS die layer stack, comprising the actual device, the bottom wafer and the cover wafer bonded on top, was glued to a carrier board which allows for electrical contacts using wire bonding (Fig. 4a). The layer stack is shown in Fig. 4b. A specially designed adaptor embracing the carrier board and the MEMS device allowed for the mounting of a standardized IEC 60318-4 ear simulator (formerly IEC 60711,^18^) incorporating a condenser microphone measuring in a closed cavity of 1.26 cm^3^ (at the back end of the chip). Additionally, the adaptor ensured decoupling of the acoustic openings in the top and bottom wafers by framing the MEMS device. The front side of the chip was left open to the surroundings. Using an audio analyzer (NTI Flexus 100) and a voltage amplifier (Krohn-Hite Model 7602 M) to drive the device, the acoustic response to sinusoidal excitation (*u*_dc_ = 40 V, *u*_ac_ = 10 V_pp_) while sweeping the frequency was obtained, as shown in Fig. 4c, d. In addition, these figures also show the simulation results of the MEMS transducer and ear simulator combined, as well as of the MEMS transducer with an acoustic output impedance as specified in ref. ^19^. Details of the simulation models are supplied in the “Materials and methods” section.

For frequencies up to 1 kHz, the SPL measurement (*p*_ref_ = 20 µPa) shows a comparatively slight variation between 68 and 70 dB. In this frequency range, the quasistatic behavior of the MEMS transducer driving the acoustic load is visible. At a referential frequency of 500 Hz, the SPL was measured at 69 dB without being influenced by dynamical effects of either the transducer itself or the ear simulator. Beyond 1 kHz, these dynamical effects become visible, resulting in an increase in the SPL. Two characteristic frequencies may be distinguished, 1.7 kHz and 11 kHz. Both are attributed to the IEC ear simulator characteristics and may be estimated from equivalent circuit models (see methods and materials section). A maximum SPL of 104 dB was measured at 11.4 kHz. This is a superposition of the ear simulator resonance and the first mode transducer resonance. This first mode transducer resonance is also visible as a shoulder at a frequency of 9.1 kHz, agreeing well with electromechanical measurements of the long actuators. The contribution to sound generation from the short actuators may be neglected due to their low number, short length (cumulative length ratio 1/4) and very low deflection (*x* ∝ *l*^−2^). From the curve of the transducer with a pure cavity, it can be concluded that the system is quite strongly damped (quality factor approximately *Q* = 2). This curve shows a significantly higher SPL than the measurement for frequencies below 5 kHz. The losses in the measurements are attributed to dissipation within the ear simulator (i.e., its RCL-network).

The THD remains below 5% for frequencies up to 500 Hz, representing the quasistatic behavior of the transducer itself. An increase to <7% at half the 1.7 kHz resonance frequency of the ear simulator can be seen. The THD exhibits maximum values at a frequency of 5.7 kHz, the equivalent of the resonance of the ear simulator at 11.4 kHz as mentioned before. Again, the signature of the transducer itself is visible as a shoulder in the curve at around 4.6 kHz, i.e., half its own resonance frequency. In contrast, the THD drops at the resonances of the transducer and the ear simulator. As before, the curve of the transducer with a pure cavity load exhibits no specific peaks except for the maximum at half the actuator resonance frequency.

For comparison, pure electromechanical measurements have also been carried out using a digital holographic microscope (Lyncee Tec R-2203). By taking a specimen without cover wafer (see Fig. 3) the lateral deflection could be obtained as a function of the driving signal frequency. The measurement results reveal a quasi-static deflection of about 1.5 µm at the middle of the 2.2 mm long actuators for *u*_dc_ = 40 V and *u*_ac_ = 10 Vpp (*f* = 50 Hz). These values fit well with the simulated deflection of the clamped-clamped beams. At resonance a deflection of about 22 µm was measured. Figure 4e shows a time wise sequence of consecutive deflections of one actuator pair.

The device’s total electrical capacitance was measured as 65 pF with an impedance spectrometer (Keysight E4990A) at a frequency of 500 Hz according to DIN 60268-7. Internal losses and dissipative effects, such as hysteresis losses, eddy current losses and heat generation, may be neglected for electrostatic transducers^20^. Assuming an efficiency of 40% for an appropriate driving circuit, handling the mostly reactive load of the electrostatic transducer, this gives a calculated sensitivity of 100 dB/mW (500 Hz, 1 mW, IEC 60318-4 ear simulator).

## Discussion

The fabricated and characterized device had a maximum SPL of 69 dB at 500 Hz in a IEC-ear simulator with a significant loss of SPL after resonance at around 10 kHz. A commonly known referential frequency response of headphones is the “Harman In-Ear Target Response Curve”^21,22^, which is the result of extensive listening tests to identify the preferred frequency responses of headphones. It shows a steep decline of the SPL from 9 kHz ongoing. A similar qualitative trend may be identified in the measurement curve. Thus, the applicability of the device does not seem to be limited by the specific resonance frequency of the actuators.

The measured SPL is enough to validate the new loudspeaker concept as an audible sound reproduction device. The THD for the transducer shows values of around 4.5% without any signal preprocessing at all. From the very simple theoretical estimations for the driving regime, one would expect a THD of about 3%. The more complex and still purely linear equivalent circuit model considers several more effects, such as the frequency dependencies of the clearance and the deflection. It is then able to predict the behavior in good agreement with the measurements for frequencies below any resonance. To lower the THD values in general, i.e., below 4.4%, design optimization is proposed. This incorporates the use of mechanical and fluidic nonlinearities counteracting the inherent Coulomb nonlinearities. Additionally, setting up the transducer for its acoustic load, i.e., the ear simulator, e.g., by diversifying the resonances, will help to enhance the audio quality further.

Based on the measured values of the quasistatic deflection (1.5 µm) of the double-S bending shape, an actively swept area of approximately *A*_act_ = 1650 µm^2^ per actuator (28 in total) may be calculated. The estimated sound pressure then yields 77 dB in a 1.26 cm^3^ volume. This value arises from the geometric approach presented in the theory section neglecting any loss in the RCL network of the ear simulator. Consequently, the value is greater than the measured SPL result (69 dB) in the ear simulator with the same volume.

Assuming a width of 20 µm of the actuator (*A*_ned_ = 20 µm * 2.2 mm), a factor *r*_area_ = 3/80 may be estimated. This calculation neglects the additional required areas (e.g., bondpads, technology constraints, etc.) which would lower the factor because the effective *A*_ned_ would increase. As such, the very same factor calculated from the sound pressure yields *r*_area_ = 1/500 (*r*_fill_ = 1/500). It follows that the present proof-of-concept is in the design optimization regime. For the boundaries (chip area of about 9.3 mm^2^ and SOI thickness of 75 µm), the maximum theoretical SPL with the presented concept is roughly 123 dB in a 1.26 cm^3^ cavity. To paraphrase, a rather large improvement of 54 dB (123–69) can be expected theoretically. Practically, an improvement to the design, mostly by denser packing of more effective actuators, i.e., an increase in *r*_area_, will allow an increase in *r*_fill_ to > 1/20, thus potentially adding > 20 dB.

The work presented shows a MEMS micro speaker concept and its proof with a fabricated specimen. Several important advantages of the concept comprise CMOS compatibility including integration capabilities with driving circuitry, precision mass production capabilities, possible low area foot print for high sound quality and quantity and very low power consumption.

### Outlook

Further work focusing on SPL increase and THD decrease is being conducted. Denser packing of the actuators thanks to design optimization is expected to allow higher efficiencies, i.e., higher SPL per area footprint. The nature of the concept allows the volume of the MEMS die to be used for sound generation. This capability is extended significantly if the thicknesses of the device layer is increased. Theoretically, the layer thickness can be increased from 75 µm to values as high as 725 µm, a typical silicon wafer thickness. This would add 20 dB for the same area footprint and fill factor. This scaling is connected with significant challenges in the technology, most prominently the DRIE process, which limits the achievable aspect ratio. The concept also allows for lateral acoustic openings, thus enabling the stacking of multiple MEMS dies to increase the SPL per area. Further optimization potential regarding distortion is expected to be released, with an optimized mechanical layout which makes use of the flexural behavior due to the actuators shape.

Future work will also address the speaker system, i.e., the development of driving circuitry comprising a step-up converter, signal amplifiers and, optionally, signal processing specifically designed for the device’s needs. The challenge is to maintain the advantages of the concept regarding ultra-low power consumption. Recently, Hänsler et al. have published work on capacitive loads and highly efficient driving circuitry^23^. They describe several amplifier classes as well as an approach to regain energy from reactive power. This is also of significant importance to the capacitive driven speaker concept presented here.

## Materials and methods

### Fabrication

**Fig. 5:**
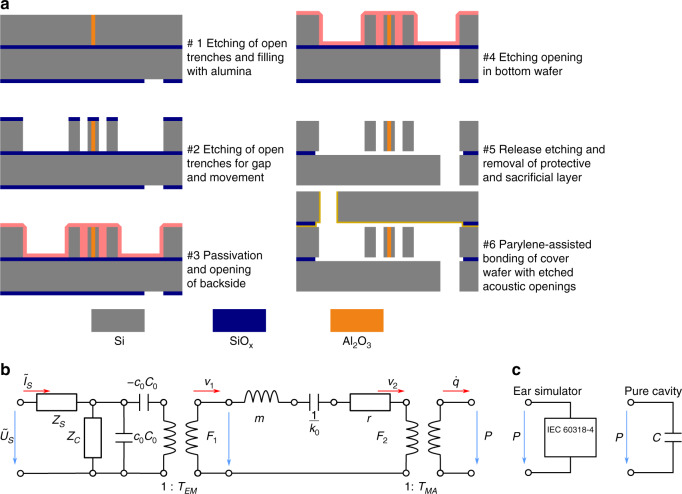
Fabrication of the device and equivalent circuit model. **a** Schematic process flow for the fabrication of the micro loudspeaker. The NED actuator shows an arrangement of three electrodes, each separated by an electrode gap. The electrodes are separated electrically by spacers made of alumina (ref. ^13^). **b** Equivalent circuit model of the micro speaker based on electrostatic bending actuators (NED). **c** Acoustic loads used. The ear simulator is taken from the literature^18^. The pure cavity condition represents a capacitor of 2.1 pF equivalent to a 297 mm^3^ volume

The MEMS micro speaker was fabricated using parylene-assisted wafer bonding, DRIE etching and atomic layer deposition (ALD). The process flow is illustrated in Fig. 5a. A 200 mm diameter, highly boron doped (10^18^ cm^−3^) bonded SOI wafer with a 75 µm device layer thickness was used as a substrate. First, open trenches were etched and subsequently filled with alumina using ALD, forming the spacers as mechanical connectors but electrical insulators in the NED actuators. Next, open trenches were etched, using DRIE again to build the actuators, anchors and the air chambers. Then, acoustic openings were etched from the backside through the handle layer (400 µm) of the bonded SOI wafer. The cover wafer was prepared by depositing and patterning the 1 µm oxide layer facing the device layer and by etching the acoustic openings. Finally, the cover wafer (400 µm) was bonded on top of the device wafer using parylene (500 nm deposited on the cover wafer, 300 °C) to assist wafer bonding^24^.

### Equivalent circuit modeling

The acoustic measurement results show the response of the MEMS transducer to the load of the IEC ear simulator. In this section, both the transducer and the IEC ear simulator are modeled using an equivalent acoustic circuit.

The equivalent circuit for the NED actuators is based on a simple plate-capacitor model presented in the literature^25^, which is transferred to an equivalent circuit according to the principles presented in^26^. The equivalent circuit model of the NED-based drive of the micro speaker comprises the electrostatic, mechanical and acoustic domains. The model is shown in Fig. 5b. In this model, the NED effect is implemented by a leveraging factor translating the plate displacement to the beam displacement, and is encoded in *T*_EM_. The actuator mass *m* is estimated from the layout. The electrical capacitance *C*_0_ and the resonance frequency were taken from measurements. The resonance frequency of 9.1 kHz was estimated from electromechanical measurements (Lyncee Tec R-2203) of the transducer without a cover wafer, taking into account the shift due to the offset voltage *u*_dc _= 40 V. Effective stiffness *k*_0_ is then calculated from resonance frequency and mass. The model is fitted using the pull-In voltage, leveraging factor (*T*_EM_) and damping coefficient *r* as fitting parameters. Pull-In voltage was fitted to 49 V as it agree with the simulation. The leveraging factor was also extracted from simulation in which a value of 51 was calculated. The difference to the fitted value of 42 is attributed to the rather complex mechanics. The damping has a substantial influence on the fitting and the fitted damping coefficient correlates to a quality factor of about 2.3. This value was qualified by acoustic measurements yielding a value of 1.5. For comparison, a quality factor of approximately 30 was calculated from the mechanical measurements in ambient atmosphere. The observed large damping in the devices with covers is attributed to the fluid dynamics of the vents and the involved flow pattern inside the 28 actuator chambers.

The equivalent circuit for the IEC ear simulator is derived from the literature^18^, and uses the parametrization published by Brüel and Kjær^19^. The pure cavity condition reflects all but one component removed from the ear simulator network. The remaining component is the capacitor as shown in Fig. 5c right. The value of the capacity is 2.1 pF, which is equivalent to a 297 cm^3^ volume of a cavity.

The models of both the transducer and the ear simulator are used to estimate the SPL and THD. The coupling of both models takes into account a certain amount of leakage between the ear simulator and the MEMS device. For the curves of the transducer with a pure cavity, presented in Fig. 4b, c, the most part of the IEC ear simulator circuit is removed. Consequently, these curves reveal behavior dominated by the MEMS transducer. The models will be presented in greater detail as well as with further development in a forthcoming publication.
